# Hypermethylation of *TRIM59* and *KLF14* Influences Cell Death Signaling in Familial Alzheimer's Disease

**DOI:** 10.1155/2018/6918797

**Published:** 2018-04-04

**Authors:** Michalina Wezyk, Magdalena Spólnicka, Ewelina Pośpiech, Beata Pepłońska, Renata Zbieć-Piekarska, Jan Ilkowski, Maria Styczyńska, Anna Barczak, Marzena Zboch, Anna Filipek-Gliszczynska, Magdalena Skrzypczak, Krzysztof Ginalski, Michał Kabza, Izabela Makałowska, Maria Barcikowska-Kotowicz, Wojciech Branicki, Cezary Żekanowski

**Affiliations:** ^1^Laboratory of Neurogenetics, Department of Neurodegenerative Disorders, Mossakowski Medical Research Centre of the Polish Academy of Sciences, 5 Pawinskiego Street, 02-106 Warsaw, Poland; ^2^Central Forensic Laboratory of the Police, 7 Aleje Ujazdowskie Street, 00-583 Warsaw, Poland; ^3^Department of Genetics and Evolution, Institute of Zoology of the Jagiellonian University, 9 Gronostajowa Street, 30-387 Krakow, Poland; ^4^Malopolska Centre of Biotechnology of the Jagiellonian University, 7A Gronostajowa Street, 30-387 Krakow, Poland; ^5^Faculty of Health Sciences, Department of Emergency Medicine, Poznan University of Medical Sciences, 10 Fredry Street, 61-701 Poznan, Poland; ^6^Center of Alzheimer's Disease of Wroclaw Medical University, 12 Jana Pawla II Street, 59-330 Scinawa, Poland; ^7^Clinical Department of Neurology, Extrapyramidal Disorders and Alzheimer's Outpatient Clinic, Central Clinical Hospital of the Ministry of Interior in Warsaw, 137 Woloska Street, 02-507 Warsaw, Poland; ^8^Laboratory of Bioinformatics and Systems Biology, Centre of New Technologies, University of Warsaw, 93 Zwirki i Wigury Street, 02-089 Warsaw, Poland; ^9^Laboratory of Bioinformatics, Institute of Molecular Biology and Biotechnology, Adam Mickiewicz University, 89 Umultowska Street, 61-614 Poznan, Poland

## Abstract

Epigenetic mechanisms play an important role in the development and progression of various neurodegenerative diseases. Abnormal methylation of numerous genes responsible for regulation of transcription, DNA replication, and apoptosis has been linked to Alzheimer's disease (AD) pathology. We have recently performed whole transcriptome profiling of familial early-onset Alzheimer's disease (fEOAD) patient-derived fibroblasts. On this basis, we demonstrated a strong dysregulation of cell cycle checkpoints and DNA damage response (DDR) in both fibroblasts and reprogrammed neurons. Here, we show that the aging-correlated hypermethylation of *KLF14* and *TRIM59* genes associates with abnormalities in DNA repair and cell cycle control in fEOAD. Based on the resulting transcriptome networks, we found that the hypermethylation of *KLF14* might be associated with epigenetic regulation of the chromatin organization and mRNA processing followed by hypermethylation of *TRIM59* likely associated with the G2/M cell cycle phase and p53 role in DNA repair with BRCA1 protein as the key player. We propose that the hypermethylation of *KLF14* could constitute a superior epigenetic mechanism for *TRIM59* hypermethylation. The methylation status of both genes affects genome stability and might contribute to proapoptotic signaling in AD. Since this study combines data obtained from various tissues from AD patients, it reinforces the view that the genetic methylation status in the blood may be a valuable predictor of molecular processes occurring in affected tissues. Further research is necessary to define a detailed role of TRIM59 and KLF4 in neurodegeneration of neurons.

## 1. Introduction

Alzheimer's disease (AD) is the most common type of dementia characterized by massive neuronal loss, primarily in the hippocampus and prefrontal cortex. Predominantly, AD is caused by the changed ratio between the long and short forms of *β*-amyloid (A*β*) peptides (A*β*40/A*β*42) and the formation of cytotoxic *β*-sheet structured oligomers resulting in progressive neuronal death and eventual loss of cognitive functions. The deposition of A*β* mono- and oligomers into senile plaques is accompanied by hyperphosphorylation of microtubule-associated protein tau forming pathological neurofibrillary tangles [1]. There are two major types of AD, early-onset (fEOAD), overlapping with familial AD (FAD), and late-onset AD (LOAD), overlapping with sporadic AD (SAD). The fEOAD represents 1–5% of all AD cases, and 40% fEOAD cases are associated with autosomal dominant mutations in *PSEN1* on chromosome 14 (encoding presenilin 1), *PSEN2* on chromosome 1 (encoding presenilin 2), and *APP* on chromosome 21 (encoding amyloid *β* precursor protein). To date, 230 mutations in *PSEN1*, 39 in *PSEN2*, and 67 in *APP* have been registered in the AD/FTD mutation database [2], including several identified by our team [3–6]. Next to the amyloid and tau pathogenesis, Alzheimer's disease involves several other pathological processes, including inflammatory states, oxidative stress, and cell cycle reentry of postmitotic neurons leading to their death [7].

Clearly, pathogenesis and etiology of Alzheimer's disease depend on the complex genetic and environmental background. This demands fine-tuned activation or repression of gene expression, and its imbalance may favor pathological conditions, including neurodegeneration [8]. Global DNA hypomethylation in neurons has been described already in the cerebral cortex of AD patients [9, 10]. Moreover, several variants of the methylation pattern of AD-related genes (e.g., *PSEN1*, *APP*) have been identified in the brain tissue of AD patients. On the other hand, a recent analysis of genes involved in the production of A*β* (*PSEN1* and *BACE1*), DNA methylation (*DNMT1*, *DNMT3A*, and *DNMT3B*), and carbon metabolism (*MTHFR*) showed no differences in their methylation status in blood DNA of LOAD patients or healthy controls [11]. Similarly, no methylation of *PSEN1* and *APP* has been found in different brain tissues of fEOAD patients [12]. In contrast, the promoter of *MAPT* was hypomethylated in a brain region-specific manner in fEOAD patients [13]. Also, the promoter of *PIN1*, encoding a protein involved in cell survival, cell cycle, and protein ubiquitination, was hypomethylated and expressed at higher levels in AD, while in frontotemporal dementia, it was hypermethylated and expressed at lower levels [14]. Other studies demonstrated an increased global DNA methylation in LOAD subjects [15]. Overall, methylation studies in AD patients have recently been collected in an excellent review by Qazi and colleagues [16]. One of the most latest studies demonstrated a hypomethylated region of the BRCA1 promoter in AD postmortem brains accompanied by an upregulation and cytoplasmic mislocalisation of the BRCA1 [17], which fully agrees with our recent results pointing to BRCA1 as the central player in DNA damage response- (DDR-) related pathology in Alzheimer's disease [18]. Finally, the existing epigenome-wide association studies (EWAS) did not so far pointed to the role of *KLF14* and *TRIM59* hypermethylation in AD. The existing EWAS data has so far described general hypomethylation, differential methylation of selected genes, for example, *S100A2*, *ALPPL2*, and *MYO1G*, or the altered content of histones and histone deacetylases (HDAC) [19–23].

Listed above data underline the importance of epigenetic modifications in AD pathology, which vary depending on the tissue or AD subtype. These data indicate the need to search for epigenetic molecular markers in relation to the type or stage of the disease. Importantly, age-associated changes in DNA methylation may regulate gene activity in developmental processes, making them accurate markers of pathological aging in AD [24]. It has been shown that hypermethylation of age-associated genes results in their general low gene expression [25]. It should be emphasized that the relationship between DNA methylation and gene expression is not straightforward, as the high transcription level can be accompanied by an elevated methylation over the gene body while hypermethylation of promoters usually leads to gene silencing [26]. Notably, it has been demonstrated that CpG sites, the methylation of which is correlated with a gene expression depending on DNA sequence variation, associated histone marks, and chromatin accessibility, play an important role in both brain development and brain disorders [27]. Finally, several reports demonstrated an influence of the methylome on the gene expression pattern, predisposing to different disease phenotypes, including obesity [28], inflammation in cancer [29], and schizophrenia and bipolar disorders [30].

In the light of the above data, Alzheimer's disease with a still not fully recognized genetic background might largely depend on epigenetic DNA modifications influencing the regulatory elements and binding affinity of transcriptional regulators. To meet this challenge, our latest research based on fEOAD blood samples revealed hypermethylation of the promoter regions of two genes correlated with aging, *TRIM59* and *KLF14*, encoding tripartite motif containing 59 and Kruppel-like factor 14, respectively [31]. This prompted us to further investigate the potential molecular implications of the hypermethylation of the two loci. Therefore, based on the transcriptomic data of fEOAD patients, in this study, we present the genetic networks for hypermethylated *KLF14* and *TRIM59* with their downstream signaling pathways, potentially relevant in the pathology of Alzheimer's disease.

## 2. Materials and Methods

### 2.1. Ethics

The local Ethics Committee of the Department of Neurology of the Central Clinical Hospital of the Ministry of Interior in Warsaw approved the protocol of the acquisition of skin biopsies and blood samples (decision number 31/2013). A written informed consent for the study and for a publication was obtained from patients (or their legal representatives) and controls, according to the Declaration of Helsinki (BMJ 1991; 302:1194). The study was approved in compliance with the national legislation and the Code of Ethical Principles for Medical Research Involving Human Subjects of the World Medical Association and at the Institute of Cardiology in Warsaw (decision number IK-NP-0021-79/1396/13).

### 2.2. Patient Cell Lines

Primary fibroblast cell lines were derived from six fEOAD patients and sixteen healthy, age- and sex-matched donors and used for whole transcriptome profiling (Table 1) as described before [18].

For DNA methylation studies, peripheral blood collected in EDTA containing tubes from 31 fEOAD patients and 57 healthy controls was used. The healthy control group was matched to the fEOAD patients using criteria of mean age and age distribution as confirmed using nonparametric Kolmogorov-Smirnov test (Table 2). The study group of 31 fEOAD patients used for blood DNA methylation studies included the same six fEOAD patients that were used for transcriptomic profiling of the fibroblast lines as described above.

### 2.3. RNA Isolation, cDNA Library Preparation, and RNA Sequencing

RNA was extracted and prepared for sequencing as described before [18]. Briefly, total RNA was isolated from fibroblast using RNeasy Mini Kit (Qiagen) according to the manufacturer's protocol. RNA quantity and quality were estimated on Qubit 2.0 using RNA BR Assay Kit and on Bioanalyzer 2100 (Agilent) using RNA 6000 Pico Kit, respectively. RNA samples with integrity number (RIN) ≥ 8 were converted to cDNA libraries using TruSeq Stranded Total RNA with Ribo-Zero kit (Illumina) according to the manufacturer's protocol and paired-end sequenced 2 × 76 bp on a HiSeq2500 Illumina platform. At least 20 M reads per sample were obtained with mean quality score (≥Q30) >94%. The sequencing data were converted to FASTQ format.

### 2.4. Bioinformatic Analysis

FASTQ files were subjected to trimming and rRNA removal and were mapped with the STAR splice junction mapper as described before [18]. The mapped reads were counted using Subread tool [32], and the FPKM (fragments per kilobase of exon per million fragments mapped) normalization method was used to quantify transcript expression [33]. Genes differentially expressed between fEOAD patients and controls were identified using edgeR software in R Bioconductor environment [34]. Cuffdiff was used to determine differential usage of promoters, differential transcription starting sites (TSS), and differential splicing [35]. Transcripts found to be differentially expressed (fold change ≥ 2, FDR ≤ 5%, *p* value ≤ 0.01) were summarized in heatmaps, volcano plots, MA plots, and dispersion plots and were subjected to principal component analysis (PCA). Differentially expressed genes were analyzed functionally by Ingenuity Pathway Analysis (IPA) software (http://www.ingenuity.com) and Reactome tools (http://www.reactome.org). The significant canonical pathways were filtered according to IPA algorithms and −log (*p* value) at cutoff = 1.3, calculated by right-tailed Fisher's exact test. *z* score was calculated according to the IPA algorithm.

### 2.5. DNA Methylation and Statistics

Total DNA was extracted from blood samples using a standard salting out procedure. Five CpG sites in *ELOVL2*, *C1ORF132*, *KLF14*, *TRIM59*, and *FHL2* were analyzed using pyrosequencing. 1-2 *μ*g of DNA was subjected to bisulfite conversion using the EpiTect 96 bisulfite conversion kit (Qiagen, Hilden, Germany) and was determined using previously applied PCR, and sequencing protocols were used to measure the DNA methylation status of the studied cytosines of interest [36]. DNA methylation percentage measured for five age-related CpG sites in gene promoter regions (ELOVL2_C7 chr6: 11044634, C1orf132_C1 chr1: 207823681, TRIM59_C7 chr3: 160450199, KLF14_C1 chr7: 130734355, and FHL2_C2 chr2: 105399288) was compared between patients and controls using independent sample Student's *t*-test.

## 3. Results and Discussion

Previously implemented analysis of DNA methylation of five age-associated genes revealed hypermethylation in the promoter regions of *TRIM59* at C7 and *KLF14* at C1 in fEOAD patients compared to healthy controls [31, 36]. In this report, we tested whether the indicated genetic status of *KLF14* and *TRIM59* could correlate with the transcriptomic profile of fEOAD patients and on which downstream processes may have an impact. For this purpose, whole transcriptome data from fibroblast cell lines were tested for differential gene expression (DGE) using edgeR, differential usage of transcription starting sites (TSS), and splicing using Cufflinks. The analysis revealed 2654 differentially expressed genes, 571 TSS, 23 promoters, 22 splicing, and 231 isoforms, listed in Supplementary Table 1. Further functional in silico analysis of DGE showed wide-range disturbances in cell cycle checkpoints and DDR pathways in fEOAD patients, which were confirmed in our previous report at biochemical and molecular biology levels in fEOAD patient-derived fibroblasts and neuronal cell lines [18]. At this point, it is worth emphasizing that by using various tissues from the same group of fEOAD patients, we were able to indicate common biological processes extracted in transcriptomic data and related to the found hypermethylated genes. This use of various tissues strengthens the obtained biological conclusions, which may indicate a more general mechanism of disease and therefore a marker, rather than only tissue specific. On the other hand, we are aware of the limitations of these studies and the need to investigate whether aberrant methylation of *KLF14* and *TRIM59* occurs in AD neurons. It is worth noting that one of the guidelines in AD research is to identify markers from the readily available tissue, that is, blood, to predict pathological mechanisms in the brain tissue, access to which is difficult during the patient's lifetime.

The top altered biological processes based on transcriptome profile were related to “*Control of Chromosomal Replication*,” “*Role of CHK Proteins in Cell Cycle Checkpoint*,” “*Mitotic Roles of Polo-Like Kinase Signaling*,” “*Role of BRCA1 in DNA Damage Response*,” “*ATM Signaling*,” “*G2/M DNA Damage Checkpoint Regulation*,” “*Estrogen-Mediated S-Phase Entry*,” “*BRCA1 Cancer Signaling*,” “*DNA Damage-Induced 14-3-3σ Signaling*,” “*DNA Double-Strand Break Repair by Homologous Recombination*,” and “*Cyclins and Cell Cycle Regulation*.” Importantly, the top enriched biological processes assigned to differentially expressed TSS were related to similar clusters, including “*Cell Cycle Control of Chromosomal Replication*,” “*G2/M DNA Damage Checkpoint Regulation*,” “*Role of BRCA1 in DNA Damage Response*,” and “*Protein Ubiquitination Pathway*,” as well as significantly enriched “*Major and Minor mRNA Splicing*.” Discrepancies at the start of transcription in the abovementioned processes suggest that epigenetic modifications affect the found TSS themselves.

**Figure 1:**
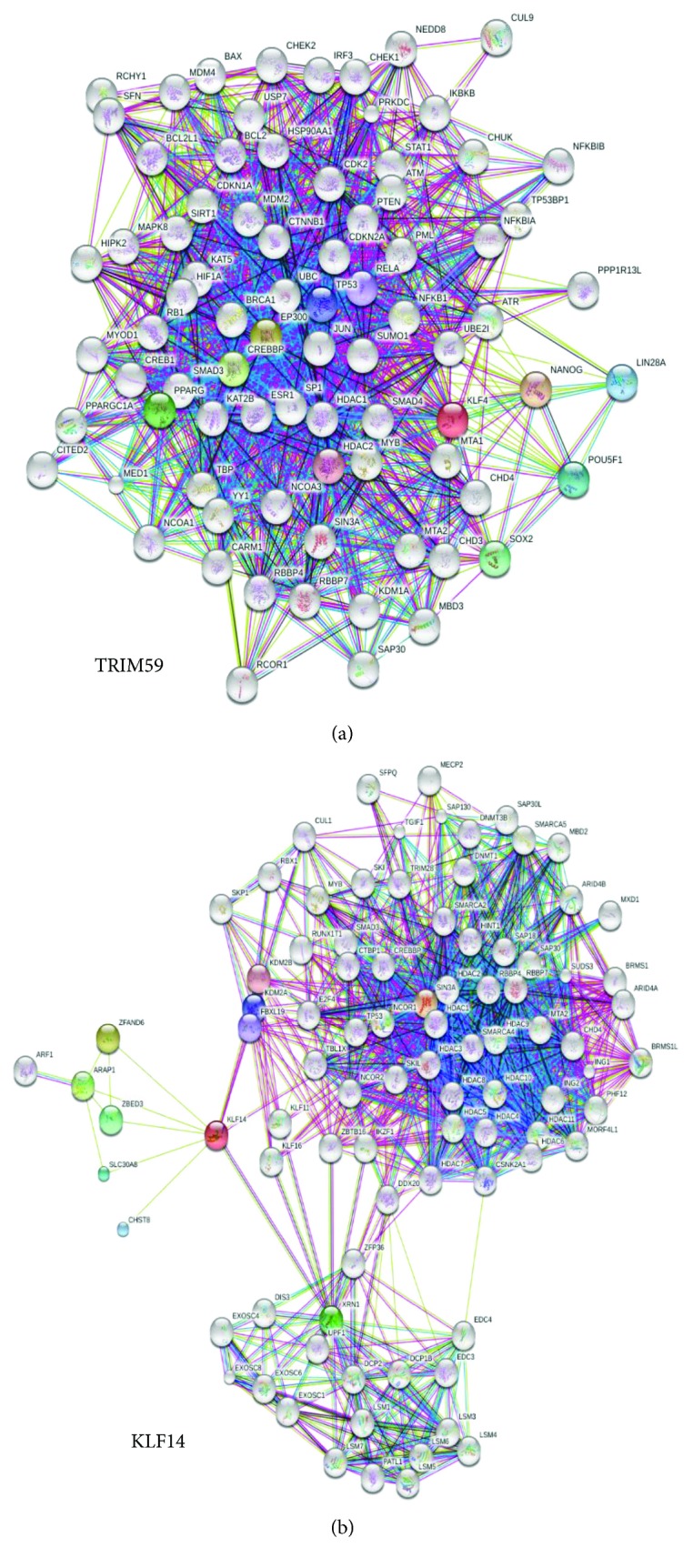
Search Tool for the Retrieval of Interacting Genes/Proteins (STRING) networks for *TRIM59* and *KLF14*. Based on “*evidence type of interaction comparison*” applied in STRING, we extracted the dataset of 2718 genes/proteins in the functional network of *TRIM59* (a) and the dataset of 2004 genes/proteins in the functional network of *KLF14* (b).

**Figure 2:**
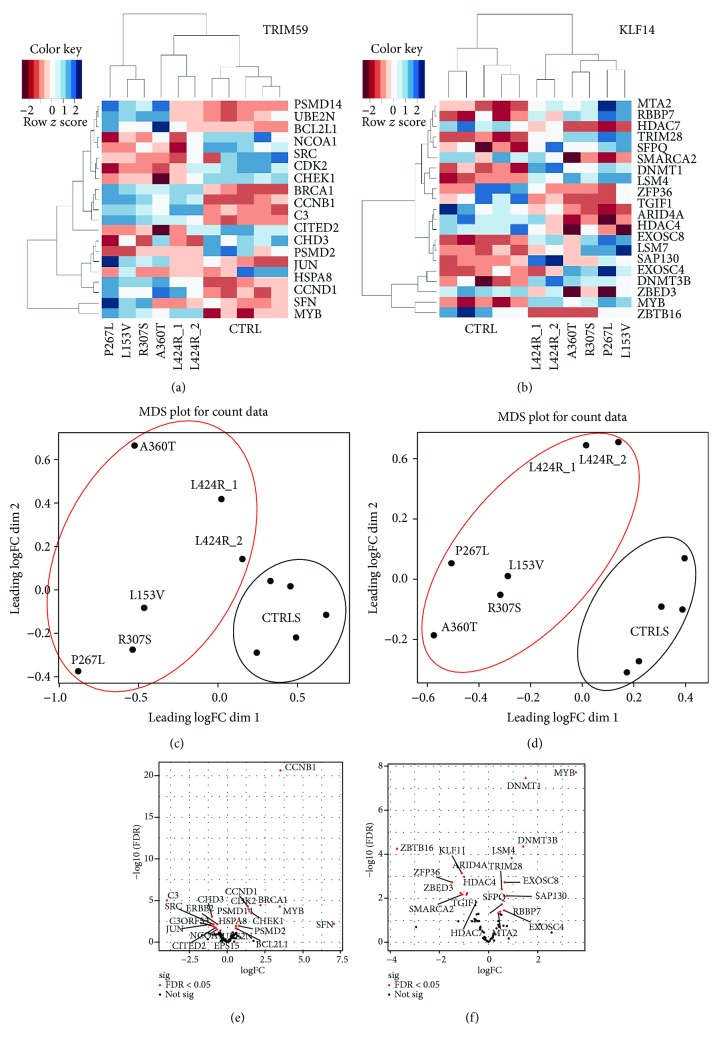
Differential gene expression analysis in *TRIM59* and *KLF14*. The *KLF14* and *TRIM59* networks contained 21 differentially expressed genes each and were visualized on the heatmaps (a, b) and volcano plots (c, d). Multidimensional scaling analysis of the networks revealed high level of specificity of individual genes in the two compared datasets of fEOAD and controls, which is highlighted in red and black circles on the graphs (e, f).

Thus, we further assessed the correlation between the methylation status of age-related genes, *KLF14* and *TRIM59*, and the whole transcriptome profile of fEOAD patient cell lines. We asked whether the hypermethylation of *KLF14* and *TRIM59* influences biological processes crucial for the development of the fEOAD phenotype. For that, we generated functional genetic networks of *TRIM59* and *KLF14* using the Search Tool for the Retrieval of Interacting Genes/Proteins (STRING) biological database. Based on “*evidence type of interaction comparison*” applied in STRING, we extracted 2718 genes/proteins in the functional network of *TRIM59* and 2004 genes/proteins in the functional network of *KLF14* (Figures 1(a) and 1(b)). Both networks contained 21 differentially expressed genes found in fEOAD transcriptomes, as visualized in the heatmap (Figures 2a and 2(b)) and volcano plots (Figures 2(c) and 2(d)). Multidimensional scaling analysis of these networks confirmed high level of specificity of individual genes in the two compared datasets of fEOAD and controls, as highlighted by the circles on the graphs (Figures 2(e) and 2(f)). The differentially expressed genes of the *TRIM59* and *KLF14* networks (Supplementary Table 1) were subjected to the functional analysis using IPA and Reactome. This analysis revealed that the DEGs of the *TRIM59* network were enriched in signaling pathways related to the cell cycle and DDR disturbances similar to those described above, while the *KLF14* network was enriched in biological processes connected with the regulation of gene expression, including chromatin organization, mRNA processing, splicing, maintenance of mRNA stability, and mRNA decay (Supplementary Table 2).

**Figure 3:**
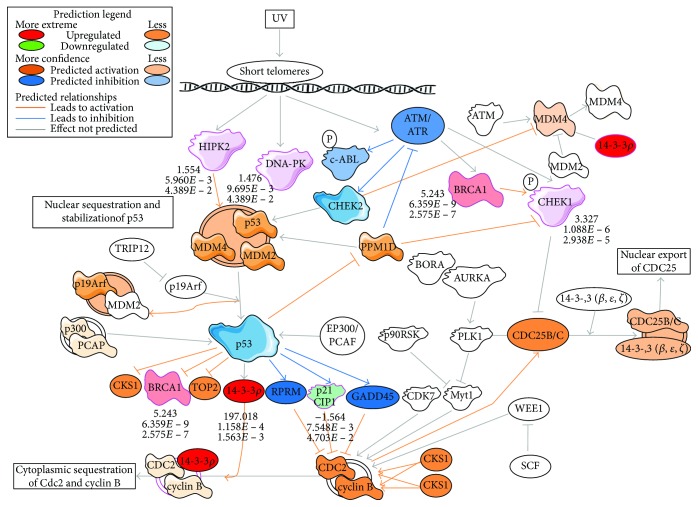
DNA damage stress response in the TRIM59 network. The pathway with upregulated or downregulated components has been extracted using Ingenuity Pathway Analysis (IPA), and the activation or inhibition of mutual relationships between the components was predicted by IPA algorithms.

Overall, the *TRIM59* network consisted of several key players of the cell cycle regulation (cyclin B1, cyclin-dependent kinase 2, and cyclin D1), proapoptotic signaling (BCL2-like 1), DNA damage response (BRCA1 and checkpoint kinase 1 (Chk1)), and proteasomal ubiquitination system (ubiquitin-conjugating enzyme E2N, proteasome 26S subunit, non-ATPase 2 and 14, and BRCA1). This is in agreement with our latest functional study showing molecular mechanisms of cell death in fEOAD fibroblasts and neurons with an underlying role of overactive and subcellularly mislocalized BRCA1 in both abnormal DNA damage response and improper turnover of presenilin 1, the key protein in amyloid pathology in AD [18]. Remarkably and consistently with our data, it has been recently suggested that the observed hypomethylation of BRCA1 in AD brains might adversely affect BRCA1 functions, leading among others to its upregulation and cytosolic relocation in AD brain [17]. Presented in this report, the TRIM59 network was enriched in abnormally activated elements of the cell cycle checkpoint and the DDR process with the leading role of BRCA1 (Figure 3). The altered methylation of TRIM59 could influence the content and functions of DDR elements, including BRCA1.

As mentioned, we found that the *KLF14* network consisted of a number of the key epigenetic regulatory proteins, including DNA (cytosine-5-)-methyltransferase 1 (DNMT1), DNA (cytosine-5-)-methyltransferase 3 beta (DNMT3b), Sin3A-associated protein (SAP3A), histone deacetylase 7 (HDAC7), histone deacetylase 4 (HDAC4), and components of exosomes involved in the transport of microRNAs. The composition of the *KLF14* network suggested its contribution to modifications of the gene architecture and gene expression machinery. Thus, *KLF14* hypermethylation could drive the dysregulations in the cell cycle and DNA damage and repair downstream *TRIM59*, as discussed below.

**Figure 4:**
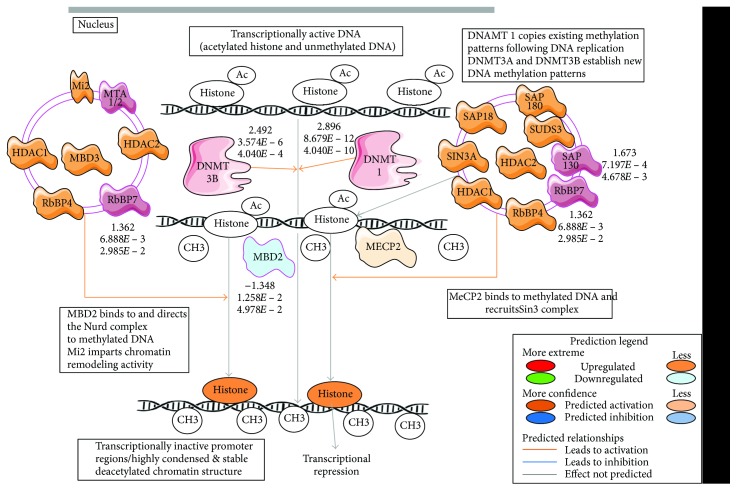
DNA methylation and transcriptional repression signaling in the *KLF14* network. The pathway with upregulated or downregulated components has been extracted using Ingenuity Pathway Analysis (IPA), and the activation or inhibition of mutual relationships between the components was predicted by IPA algorithms.

*TRIM59* and *KLF14* networks revealed a different enrichment in the biological pathways. The network of hypermethylated *KLF14* in fEOAD patients was distinguished by the processes responsible for epigenetic regulation of gene expression, including chromatin organization and modifications, maintenance of mRNA stability, and mRNA decay, as well as regulation of mRNA splicing. Hypermethylated KLF14-driven destabilization in the machinery responsible for the maintenance of genome architecture was in agreement with differential usage of TSS and differential mRNA splicing estimated by Cufflinks with a Cuffdiff mode in fEOAD transcriptomes. According to the Reactome-based enrichment analysis, the differential usage of TSS in fEOAD was assigned to altered major and minor pathways of the mRNA splicing. These data suggest that hypermethylation of *KLF14* could affect the chromatin architecture and gene expression pattern in Alzheimer's disease. In addition, we found that the network of hypermethylated *KLF14* was enriched in signaling pathways regulating DNA methylation and transcription repression (Figure 4). Recently, it has been reported that *KLF14* associates with H3K9me3 histone marks and with the corresponding histone methyltransferase complex, contributing to the reshaping of T cell fate by influencing their differentiation program [37]. Moreover, the *KLF* family was found to be involved in the transcriptional modulation of neuronal genes, for example, dopamine D2 receptor [38]. On the other hand, *KLF14* reduction was reported to be responsible for an abnormal centrosomal amplification and aneuploidy [39]. This suggests that *KLF14* could be involved in modulation of gene expression in neurons, leading to neuronal cell cycle reentry and an abnormal DNA damage response under pathological conditions in Alzheimer's disease [40].

**Figure 5:**
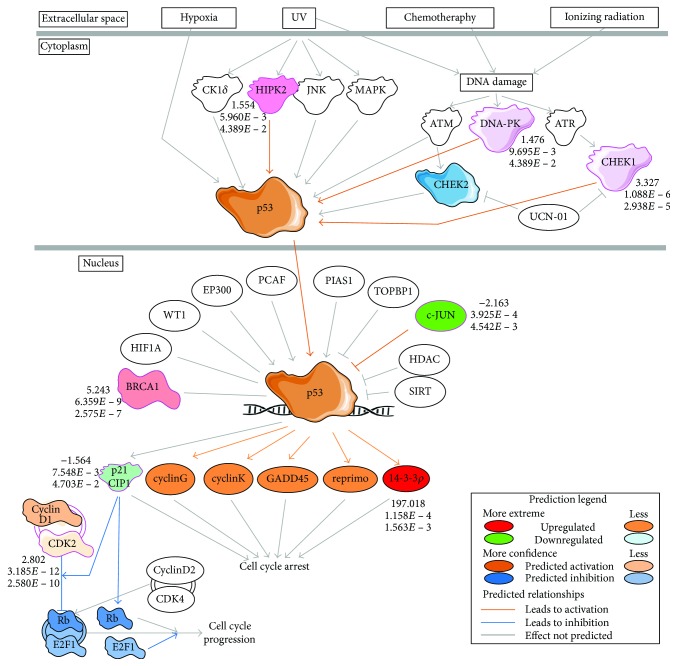
p53 signaling in the *TRIM59* network. The pathway with upregulated or downregulated components has been extracted using Ingenuity Pathway Analysis (IPA), and the activation or inhibition of mutual relationships between the components was predicted by IPA algorithms.

Opposite to *KLF14*, the network of hypermethylated *TRIM59* in fEOAD patients was enriched in the processes related to cell cycle regulation, including cell cycle phase checkpoint regulation (mainly G2/M) and p53-dependent regulation of transcription of DNA repair genes as well as ubiquitin-dependent degradation of cyclins. These data suggested separated mechanisms between *KLF14* and *TRIM59* hypermethylation in AD, despite the fact that both may potentially contribute to the accelerated pathological aging. The networks could cooperatively lead to a loss of genome integrity and cell death, and *KLF14* could play an overriding role and provide an upstream mechanism in this process. Furthermore, *TRIM59* which encodes an ubiquitin ligase might be involved in the affected proteostasis in the neurodegeneration process, for instance, by contributing to the accumulation of the neurofilament light chain, similar to *TRIM2* [41]. In turn, other data suggested a proapoptotic cooperation of p53 and TRIM59, where upregulation of *TRIM59* resulted in ubiquitination and degradation of p53 [42]. Consistently, our studies suggested that hypermethylation-driven inhibition of *TRIM59* expression in fEOAD could indeed correspond to the increased activation of p53 observed in fibroblasts and neurons [18]. This conclusion is based both on the in silico bioinformatic analyses with IPA and Reactome tools performed for the given RNA-seq dataset (Figure 5); it is supported by the observed increase in the level of p53 protein phosphorylated at Ser15, especially upon DNA damage induction [18]. Our results suggest that hypermethylation of *TRIM59* might play a role in proapoptotic signaling in AD mediated by p53. In addition to p53, the majority of altered canonical pathways in the *TRIM59* network were related to DDR that was shown by RNA-seq-based IPA prediction of the destabilization of the ATM-Chk1-BRCA1-p53 axis, with upregulation of BRCA1 and Chk1 in fEOAD patients (Figure 3), which is in agreement with our other data validating these predictions at protein levels [18]. Importantly, the p53 transcription factor is a key regulator of senescence via different mechanisms, including nuclear lamin defects that activate p53 and induce expression of the target genes of p53 [43], induction of DDR and telomere shortening, or posttranslational regulation of p53-mediated DDR [44]. Based on the above, we suggest that *TRIM59* and the methylation status of its promoter region could constitute a molecular switch between biological and pathological aging via the p53 pathway.

## 4. Conclusion

Overall, the hypermethylation pattern of the promoter regions of *TRIM59* and *KLF14* in fEOAD patients might contribute to genetic instability in fEOAD patients. To eliminate any limitations rising from tissue differences, hypermethylation of *TRIM59* and *KLF14* should be tested in the future in neurons derived from patients with fEOAD. Nevertheless, our data obtained for different tissues provide the view that the DNA methylation pattern in promoters of *KLF14* and *TRIM59* in blood can be used not only as a predictor of age but also as a marker of specific molecular pathomechanisms present in AD neuronal cells, an example of which is abnormal signaling of DNA damage. Summarizing, based on the above, our results suggest that hypermethylation of *KLF14* and *TRIM59* might contribute to cell death and progression of Alzheimer's disease accompanied by accelerated and premature aging.

## Figures and Tables

**Table 1:** Characteristics of the tested groups used for RNA-seq.

Tested groups	*N*	Mean age ± SD	Min age	Max age	Male (%)
Healthy controls	16	41.1 ± 20.2	41	81	50
fEOAD patients	6	47 ± 10.7	31	67	50

**Table 2:** Characteristics of the tested groups used for methylation studies.

Tested groups	*N*	Mean age ± SD	Min age	Max age	Male (%)
Healthy controls	57	46.44 ± 10.5	28	66	63.2
fEOAD patients	31	44.2 ± 10.2	31	68	48.4

## Supplementary Materials

Supplementary 1 Table 1: analysis of differentially expressed genes (DEGs) of six familial early-onset Alzheimer's disease patients with *PSEN1* mutations versus sixteen neuropsychological healthy age- and sex-matched controls. RNA was isolated from established fibroblast cell lines and sequenced as described in Materials and Methods. DGE analysis performed by edgeR software revealed 2654 DEGs (“EDGER_biotype 8EOADv.16CTRL”_excel sheet), 21 DEGs exclusive for the KLF14 network (“KLF14_edger_biotype”_excel sheet), and 21 DEGs unique for the TRIM59 network (“TRIM59_edger_biotype”_excel sheet), and analysis performed with Cufflinks revealed 571 transcription starting sites (“TSS”_excel sheet), 23 promoters (“PROMOTERS”_excel sheet), 22 splicing (“SPLICING”_excel sheet), and 231 isoforms (“ISOFORMS”_excel sheet).

Supplementary 2 Table 2: enrichment of canonical signaling pathways in familial early-onset Alzheimer's disease patients compared with controls. KLF14 and TRIM59 DEG datasets from Supplementary Table 1 were subjected to functional enrichment analysis with IPA (“KLF14_IPA”_sheet and “TRIM59_IPA”_sheet) revealing lists of canonical pathways, upstream regulator, diseases and biofunctions, Tox functions, and regulator effects. The −log of *p* value was calculated by right-tailed Fisher's exact test, and the cutoff of 1.3 was applied, meaning that pathways with a *p* value equal to or lower than 0.05 are displayed. The *z* score ≤ 1 identified functions with the strongest predictions for a drop of the activity of the tested signaling pathway and *z* score ≥ 1 identified functions with the strongest predictions for an increase in the activity of the tested signaling pathway. The enrichment analysis was also performed with the Reactome online tool (“KLF14_REACTOME” and “TRIM59_REACTOME”_sheet), sorted by “Entities pValue” with statistical significance at *p* ≤ 0.05 level, revealing that the TRIM59 network was enriched in signaling pathways related to the cell cycle and DDR disturbances, while the KLF14 network was enriched in biological processes related to the regulation of gene expression, chromatin organization, mRNA processing, splicing, maintenance of mRNA stability, and mRNA decay.
